# Partial Sigma Covalent Bonding in Transition Metals

**DOI:** 10.1002/jcc.70373

**Published:** 2026-04-29

**Authors:** Lam H. Nguyen, Thanh N. Truong

**Affiliations:** ^1^ Department of Chemistry University of Utah Salt Lake City Utah USA

**Keywords:** d^8^–d^8^ transition‐metal interaction, lantern organic frameworks, orbital reordering, partial sigma covalent bonding

## Abstract

This work establishes partial σ‐covalent bonding as a general electronic phenomenon extending from main‐group biradicals to d^8^–d^8^ transition‐metal systems (Co(I), Rh(I), Ir(I)). Using dispersion‐corrected DFT (B3LYP‐D3/def2‐TZVP for transition metals and 6‐31+G(d) for other elements) in combination with Wiberg bond index and frontier molecular orbital analyses, we show that partial σ‐bonding is strongly governed by ligand field and orbital symmetry. While shorter metal–metal distances correlate with larger bond orders, singlet–triplet energetic differences arise from competition between ligand‐field splitting and exchange energy and spin‐orbit coupling. Compared to isolated metal–metal ion dimers at the same distance, both ligand types in the study modify not only the orbital characters but also the frontier orbital energy levels. Strong field C‐donor ligands significantly widen the HOMO–LUMO gap while changing orbital ordering so that the HOMO has d_z_^2^–d_z_^2^ antibonding character and the LUMO has a bonding p_z_–p_z_ orbital. Consequently, it leads to triplet‐dominant metal–metal bonding, (WBO_Triplet_ = 0.4; *d*_M–M_ = 2.9 Å). In contrast, N‐donor lantern organic frameworks (LOFs) narrow the HOMO–LUMO gap while alternating orbital ordering so that the HOMO becomes the ligand‐based bonding π‐orbital and the LUMO corresponds to a d_z_^2^–d_z_^2^ antibonding orbital, thereby enabling substantial σ‐bonding in both spin states (WBO_Triplet_ = 0.3 with *d*_M–M_ = 3.1 Å; and WBO_Singlet_ = 0.7 with *d*_M–M_ = 2.7 Å). More importantly, N‐donor LOF environments significantly reduce the HOMO–LUMO gap up to 2.00 eV relative to comparable conventional systems, suggests a viable strategy for band gap engineering at the single‐unit‐cell level, without requiring infinite stacking.

## Graphical abstract

This study demonstrates partial σ‐covalent bonding is a general electronic phenomenon spanning main‐group biradicals and d^8^–d^8^ transition‐metal systems (Co, Rh, Ir). The analyses in this study reveal that ligand field (Dimen and Lantern organic frameworks) and orbital symmetry govern metal–metal interactions, with different bonding expressions.

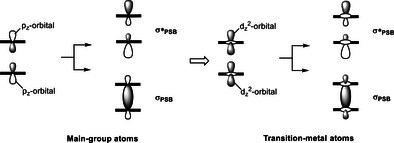

## 1 Introduction

In conventional chemistry, a sigma covalent bond arises from the sharing of two electrons in molecular orbitals originated from *head‐to‐head overlap* of atomic orbitals from the two atomic centers, resulting in a bond order of 1.0. A fundamental question has been raised: *Can a partial σ‐bond exist? In other words, can σ*‐*bond have a bond order lower than one?* Recent findings indicate that it is a possibility though yet waiting for direct experimental confirmations. One of the earliest experimental hints came from the work of Michl and co‐workers [1], who observed that the singlet state was more stable than the triplet in the cubane biradical C_8_H_6_, implying a weak σ‐type interaction between the radical centers. Subsequent experimental studies by Schnepf and colleagues on the germanium cubane analogue [Ge_8_(N(SiMe_3_)_2_)_6_] confirmed the presence of a stably isolated singlet state [2, 3]. On the other hand, a quantum chemistry study of this cubane‐like framework by Pan et al. [4] revealed a diagonal Ge–Ge connection with shared‐electron values markedly lower than those of a typical Ge—Ge covalent bond, with Wiberg bond order lower than 1.0, evidence of a partial σ‐covalent interaction.

More recently, computational studies by Truong and co‐workers [5, 6] further demonstrated that partial σ‐bonds could form between two carbon atoms and provided a detailed analysis on the nature of such bonds. In particular, these bonds were stabilized from molecular orbitals arising from p_z_–p_z_ head‐to‐head overlap with Wiberg bond orders of 0.38–0.48 and can have exceptionally long C—C bond distances (2.62–5.93 Å), far beyond the longest known single C—C bond (1.81 Å in dihydropyracylene) [7]. Moreover, the frontier orbitals show an unconventional inverted pattern, in which the HOMO has antibonding character and the LUMO has bonding.

**FIGURE 1:**
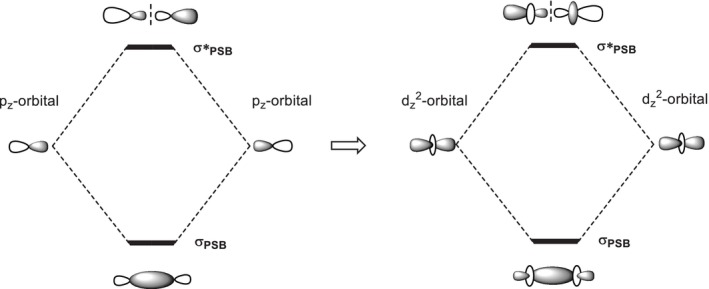
Schematic description of partial sigma bond (PSB) molecular orbitals from p_z_–p_z_ overlap to d_z_^2^–d_z_^2^ overlap.

It is clear the next question to ask is whether such unconventional bonding extends beyond main group systems, such as between transition metal atoms. If taking a simplified model as shown in Figure 1, then the question would be the head‐to‐head overlap between d_z_^2^ atomic orbitals forming partial σ‐bonding similar to those seen in the main group?

It is interesting to note that an early experimental study of Gray and co‐workers [8] found that Rh(I) complexes with Rh–Rh interactions far beyond the covalent range, stabilized by head‐on d_z_^2^–d_z_^2^ and p_z_–p_z_ derived orbital overlap. Complementing this experimental observation, a theoretical study of Novozhilova et al. [9] revealed that in the complex [Rh_2_(1,3‐diisocyanopropane)_4_]^2+^, the triplet state actually showed a higher Rh—Rh bond order than the singlet. This phenomenon is not exclusive to rhodium; analogous interactions are observed in Ir(I) and Pt(II) complexes, confirming that this orbital behavior seems to be a general characteristic of d^8^–d^8^ electronic configurations [10, 11].

At the first glance, these findings may be a sharp contrast with carbon biradical cages, but the insight is more complicated. In the main group systems, partial σ‐bonding arises from the singlet ground state [5, 6], whereas in transition metal systems, partial bonding occurs in both spin states, with the excited triplet possessing a higher bond order than the singlet, which is anomalous from the conventional bonding theory [9]. While main group biradical systems derive both their HOMO and LUMO purely from their atomic constituents, p_z_ orbitals, previous studies indicate that transition metal complexes have a distinct orbital separation, where the HOMO is typically dominated by d_z_^2^ character and the LUMO by metal p_z_ character [11].

These results indicate that the partial σ‐bonding theoretically exists between transition metals; however, current understanding on the nature of partial σ‐bonds in transition metal complexes remains fragmented. Existing studies typically provided isolated specific characteristics, such as bond length, bond order, spin state energetics, or orbital inversion, and focus narrowly on specific metals like Rh or Ir, coordinated primarily by Dimen (1,8‐diisocyanomenthane) or its C‐donor derivatives [9, 12, 13, 14, 15, 16, 17, 18]. The focus has been on a systematic comparison across different metal centers, such as in a study of R. Mann and coworkers in a comparison of Rh and Ir in Dimen‐based complexes in different counter ion effects [10]. It is well known from the Ligand Field Theory that ligands can affect the nature of HOMO and LUMO orbitals and their gap [19]. For instance, several cage‐like transition metal complexes such as [Pt_2_(μ‐P_2_O_5_H_2_)_4_]^4−^, [Pt_2_(μ‐P_2_O_5_(BF_2_)_2_)_4_]^4−^, and [Rh_2_(1,3‐diisocyanopropane)_4_]^2+^ complexes [9, 20, 21] display the HOMO has antibonding character while LUMO has bonding. However, a study of Ir(I) complexes based on 1,3‐diene‐1,4‐diyl backbone has exhibited a completely different picture, particularly its HOMO having the bonding character [14].

**FIGURE 2:**
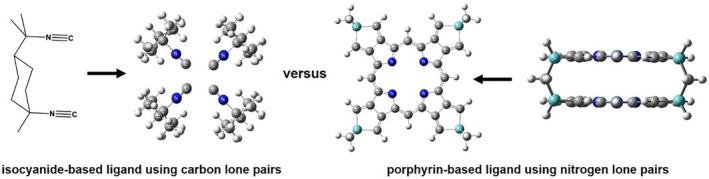
Two ligand types: isocyanide (C‐Donor) vs. porphyrin (N‐Donor) in LOFs, where white represents hydrogen atoms, gray represents carbon atoms, blue represents nitrogen atoms and aqua‐green represents the silicon atoms.

It is clear that there is a need for systematic investigation on the nature of partial σ‐bond in transition metal complexes, with a special focus on the effects of different ligands. This is the goal of this study. In particular, we employed two complementary ligand platforms: (1) previously used in an experimentally studied ligand, *Dimen* with C lone pair‐donors and (2) a theoretically designed platform porphyrin‐based Lantern Organic Frameworks (LOFs) [22, 23, 24], with N lone pair donors, as shown in Figure 2. LOF structure can be established as a common ligand framework analogous to *Dimen*, and thus allow a comparison of the difference in the potential from the two ligands. Moreover, these cage‐like geometries impose well‐defined metal–metal distances, creating a suitable structural platform to probe the distance dependence of partial σ‐bonding. Finally, we selected the metal elements from the same group Co(I), Rh(I), and Ir(I), to systematically investigate periodic trends, specifically how the increase in principal quantum number (3d^8^ to 4d^8^ to 5d^8^) influences overlap efficiency and bonding characters. Furthermore, these cations possess a number of existing experimental data as mentioned above, providing strong evidence for validating our theoretical analyses when possible.

## 2 Methods

**FIGURE 3:**
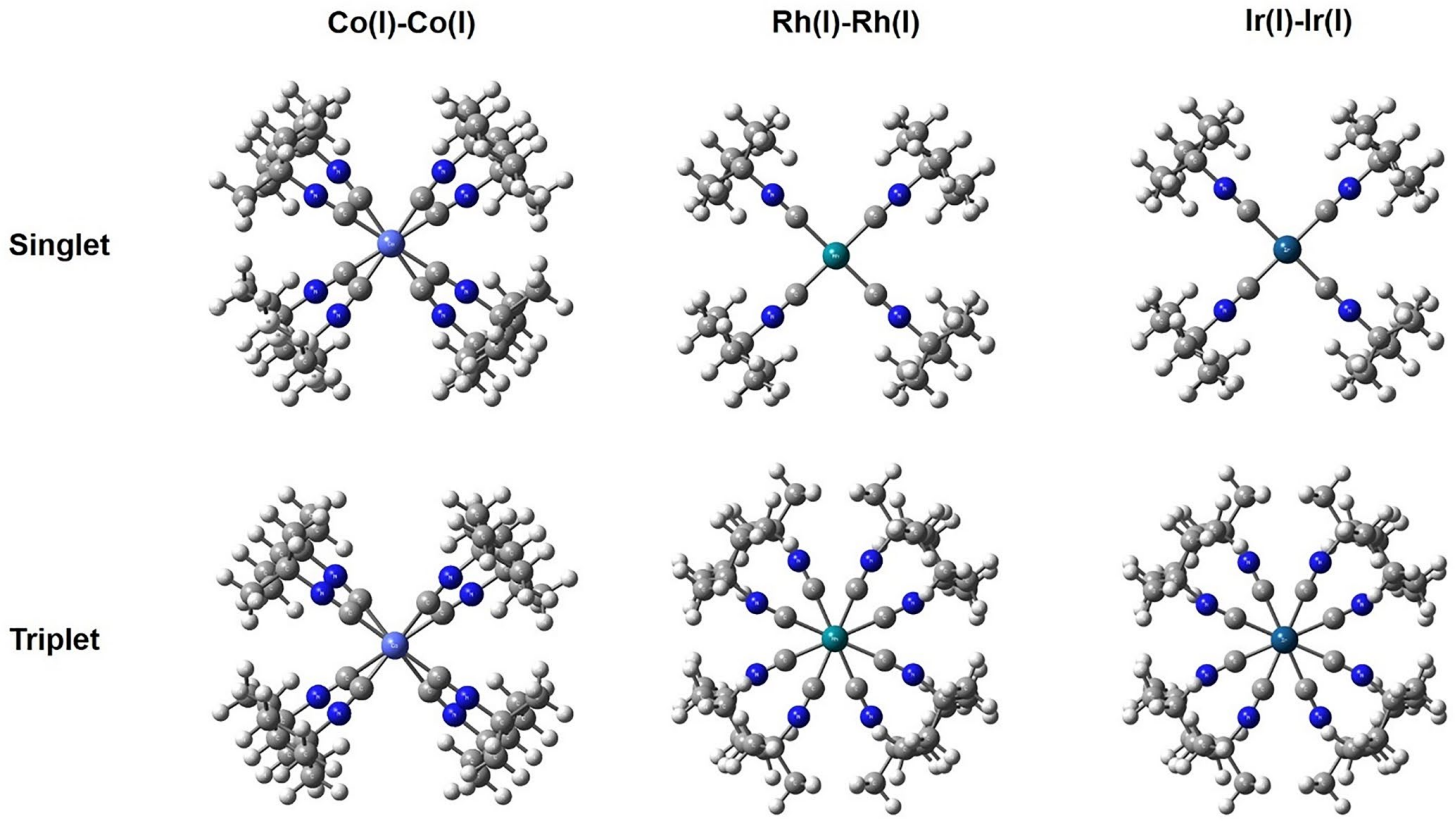
Optimized structures of Co(I), Rh(I), and Ir(I) complexes with 1,8‐ diisocyanomenthane ligand, where white represents hydrogen atoms, gray represents carbon atoms, blue represents nitrogen atoms, aqua‐green represents the silicon atoms, purple represents Cobalt atoms (Co), blue green represents Rhodium atoms (Rh) and green blue represents Iridium atoms (Ir). The ball in center of Dimen framework from top view is the considered transition metal.

**FIGURE 4:**
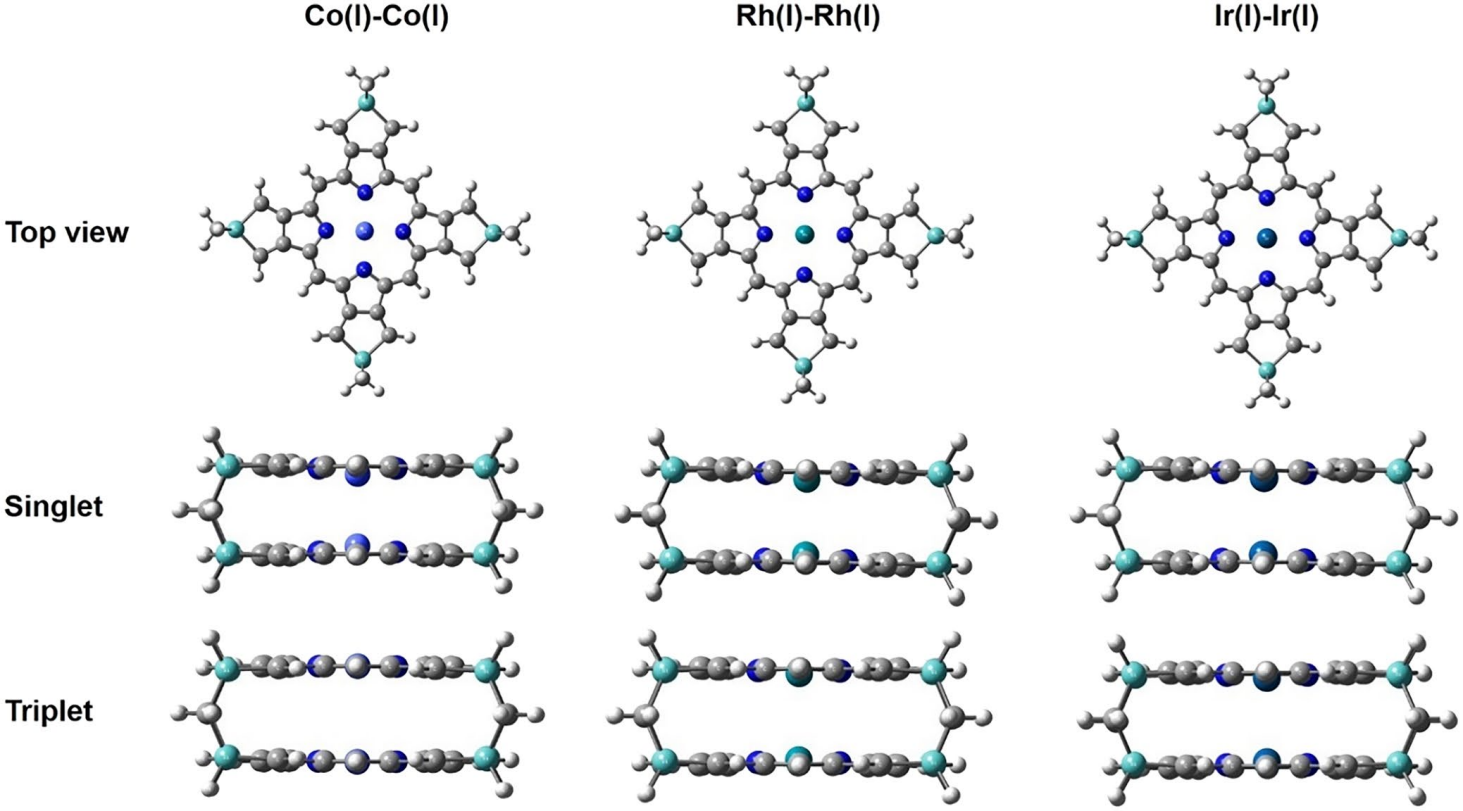
Optimized structures of Co(I), R(I), and Ir(I) complexes with (4Si‐porphyrin‐4‐methane)_2_ ligand, where white represents hydrogen atoms, gray represents carbon atoms, blue represents nitrogen atoms, aqua‐green represents the silicon atoms, purple represents Cobalt atoms (Co), blue green represents Rhodium atoms (Rh) and green blue represents Iridium atoms (Ir). The balls in center of porphyrin ring from top view are the considered transition metals.

All optimized structures shown in Figures 3 and 4 were performed by the GAUSSIAN16 program [25], at the level of theory B3LYP‐D3 [26, 27] density functional theory (DFT) including Grimme's dispersion [28, 29] interaction. The D3‐correction is important for bonds in long‐range dispersion [30, 31]. For transition‐metal centers (Co, Rh, Ir), relativistic effective core potentials (ECPs) with the def2‐TZVP [32, 33, 34] basis set were employed and 6‐31+G(d) for carbon, hydrogen, nitrogen, and silicon atoms [35, 36]. Moreover, Wiberg Bond Index [37] characteristics were examined by Multiwfn 3.8 [38].

## 3 Results and Discussion

### 3.1 Bonding Analysis

From Table 1, we first found that the shorter metal–metal partial bonds in transition metal interactions correspond directly to the larger WBO orders, which is consistent with the conventional relationship between bond length and bond order [41]. To further examine whether this correlation arises purely from geometric effects or also reflects differences in electronic states, we conducted a cross‐evaluation of bond distances and Wiberg bond indices for both singlet and triplet configurations, as summarized in Table 2. In this analysis, the Wiberg bond orders for each spin state were examined alongside the corresponding M–M distances obtained from the optimized geometries. The cross‐evaluation in Table 2 reveals that the Wiberg bond index is influenced by two competing factors: metal—metal bond distance and frontier‐orbital character. When complexes share the same electronic state, shorter M–M distances generally correspond to larger WBI values. However, when evaluated at comparable geometries, the electronic state plays a decisive role. In the Dimen series, the triplet state exhibits higher bond orders than the singlet state, whereas the opposite trend is observed in the LOF series, where the singlet state shows larger bond orders than the triplet. This comparison demonstrates that bond distance alone cannot fully account for the WBI values and that electronic structure effects must also be considered.

**TABLE 1:** Bond distances (Å), Wiberg bond orders, atomic charge distributions from NBO Analysis (*Q*_NBO_), HOMO*–*LUMO gaps at singlet state, and total energy difference between singlet and triplet states (Δ*E*_S–T_) of Co(I), Rh(I), and Ir(I) complexes.

Ligand		1,8‐diisocyanomenthane (Dimen)						(4Si‐porphyrin‐4‐methane)_2_ (LOF)					
Metal	State	Bond distance (Å)		WBO	*Q*_NBO_	Δ*E*_S–T_	HOMO–LUMO gap (eV)	Bond distance (Å)		WBO	*Q*_NBO_	Δ*E*_S–T_	HOMO–LUMO gap (eV)
		M—M	C—M					M—M	N—M				
Co(I)—Co(I)	Singlet	3.98	1.855	< 0.05	−0.06	−10.79	3.03 (0.80)^a^	2.67	2.036	0.66	+0.90	+31.97	0.89 (1.62)^c^
	Triplet	4.01	1.994	< 0.05				3.54	2.032	< 0.05			
Rh(I)—Rh(I)	Singlet	4.09^b^	1.979	0.06^c^	−0.12	−39.95	3.48 (1.27)^c^	2.73	2.077	0.64	+0.74	+3.72	1.00 (1.63)^c^
	Triplet	2.92^b^	1.983	0.39				3.08	2.068	0.30			
Ir(I)—Ir(I)	Singlet	3.97^b^	1.980	0.08^c^	−0.07	−29.21	3.13 (1.37)^c^	2.74	2.072	0.69	+0.84	−2.53	1.24 (1.57)^c^
	Triplet	2.92^b^	1.985	0.40				3.06	2.060	0.31			

*Note:* The Δ*E*_S–T_ values, where Δ*E*_S–T_ = *E*_Singlet_–*E*_Triplet_ (kcal mol^−1^), with *E*_singlet_ and *E*_triplet_ indicating the total energies at the corresponding states.

^a^ The values in the brackets are HOMO–LUMO gaps of isolated M–M at the same distance as M–M in the complex.

^b^ Within the range of metal–metal bond distances in twisted to eclipsed conformations, from 3.24 to 4.52 Å of [Rh_2_(Dimen)_4_]^2+^ and from 2.90 to 4.30 Å of [Ir_2_(Dimen)_4_]^2+^ salts [10, 12, 13, 15, 16, 18, 39, 40].

^c^ Although Wiberg bond orders greater than 0.05 to below 0.1 are sometimes reported as indicative of weak interactions, values in this range are comparable to the uncertainty of the computational method. Therefore, for the purpose of the present analysis, we adopt a stricter criterion in which WBO values below 0.10 are regarded as insignificant and are not interpreted as evidence of meaningful bonding.

**TABLE 2:** Cross‐evaluation of bond distance and Wiberg bond order relationship.

Ligand		1,8‐Diisocyanomenthane (Dimen)			(4Si‐porphyrin‐4‐methane)_2_ (LOF)		
Metal	Geometry	M—M bond distance (Å)	M—M Wiberg bond order		M—M bond Distance (Å)	M—M Wiberg bond order	
			Electronic state			Electronic state	
			Singlet	Triplet		Singlet	Triplet
Co(I)—Co(I)	Singlet	3.98	< 0.05	< 0.05	2.67	0.66	0.37
	Triplet	4.01	< 0.05	< 0.05	3.54	0.26	< 0.05
Rh(I)—Rh(I)	Singlet	4.09	0.06	0.22	2.73	0.64	0.37
	Triplet	2.92	0.32	0.39	3.08	0.42	0.30
Ir(I)—Ir(I)	Singlet	3.97	0.08	0.22	2.74	0.69	0.42
	Triplet	2.92	0.32	0.40	3.06	0.57	0.31

Moreover, clear relationships of metal charge distribution (*Q*_NBO_), HOMO–LUMO gap, and spin‐state preference that depend strongly on the ligand environment are also revealed in Table 1.

In the Dimen (isocyanide, C‐donor) complexes, the natural charges on the metal centers are slightly negative (around −0.10e), which is far away from the positive charge (+1.00e) of the isolated metal ion. This indicates substantial charge transfer between the ligand and the metal, leading to strong ligand‐field splitting and consequently large HOMO–LUMO gaps about 3.03–3.48 eV. These gaps are considerably larger than those of the corresponding isolated metal ions at comparable metal–metal distances (0.80–1.37 eV). Such large gaps favor electron pairing and stabilize the low‐spin (singlet) configuration, explaining why the singlet state is consistently more stable than the triplet in the Dimen systems, specifically with ΔE_S‐T_ values of −10.79 kcal mol^−1^ for Co, −39.95 kcal mol^−1^ for Rh, and −29.21 kcal mol^−1^ for Ir.

In contrast, metal centers in LOF (porphyrin, N‐donor) complexes retain much of their positive charge (+0.80e), indicating limited charge transfer. The N‐donors cause a weaker field compared to both C‐donors in the Dimen complexes, leading to smaller HOMO–LUMO gaps (0.89–1.24 eV). Notably, these gaps are even smaller than those of the corresponding isolated metal ions (1.57–1.62 eV). Consequently, energy gained from exchange energy and spin‐orbit coupling can compensate for the cost of populating higher energy orbitals in high‐spin states and thus can lead to the triplet state energetically preferred for Co– and Rh–LOF complexes, with the splitting of Δ*E*_S–T_ values (+31.97 and +3.72 kcal mol^−1^, respectively) as shown in Table 1. Collectively, Dimen and LOF ligands present opposite ligand field effects: the strong C‐donor isocyanide ligands cause substantial charge transfer and widen the HOMO–LUMO gap, whereas the weaker N‐donor porphyrin ligands result in minimal charge transfer and narrow the orbital gap.

Moreover, as expected from ligand field theory, the splitting energy (Δ_o_) rises with the metal's principal quantum number due to increased *d*‐orbital radial extension, such as from [Co(NH_3_)_6_]^3+^ to [Ir(NH_3_)_6_]^3+^ with Δ_o_ from 2.83 to 4.96 eV [42], is mirrored in the M‐LOF series. Here, the HOMO–LUMO gap widens down the group from 0.89 to 1.24 eV. As this gap expands, the low‐spin singlet state becomes more energetically favored. Consequently, the Ir complex exhibits a distinct singlet–triplet inversion with Δ*E*_S_–_T_ = −2.53 kcal mol^−1^.

However, this energetic inversion represents only a part of the picture. The singlet Ir—Ir bond also has a substantial partial σ‐bond order (WBO = 0.69). This value exceeds the Ir—Ir bond orders reported for other Ir‐complexes in a 1,3‐butadiene‐1,4‐diyl backbone and in metallic iridium chain crystals, where the maximum WBO reaches at most 0.47 at a comparable Ir—Ir bond distance of 2.74 Å [14, 43]. Such enhancement in WBO in the singlet state of the Ir–LOF complex deserves further analysis such as orbital analysis given below.

### 3.2 Frontier Molecular Orbital Analysis

**FIGURE 5:**
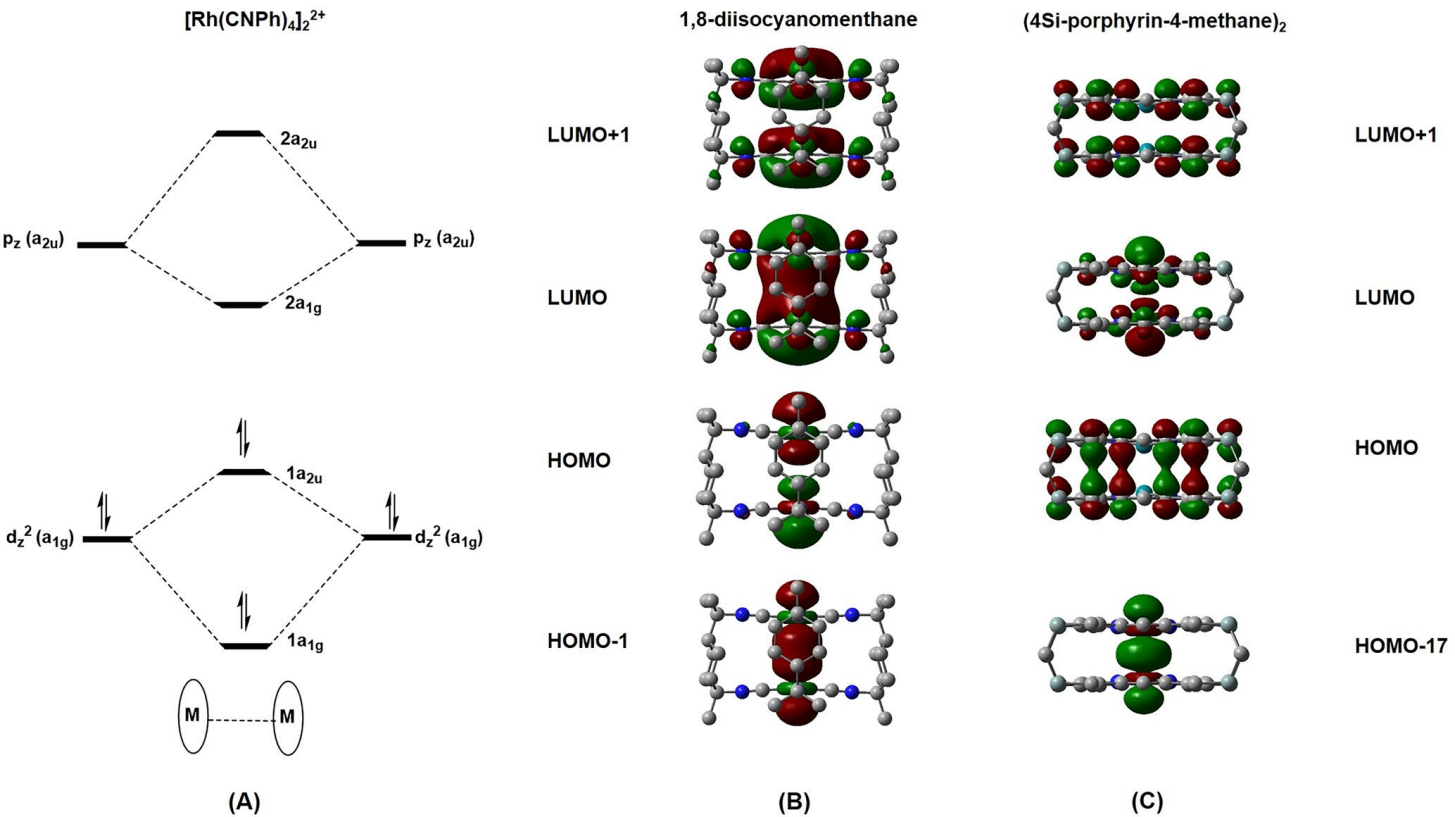
Visualization of frontier molecular orbitals of Rh(I) complexes with CNPh ligand (A), 1,8‐diisocyanomenthane ligand (B), and (4Si‐porphyrin‐4‐methane)_2_ ligand (C) at the singlet state, (isovalue = 0.02), where the hydrogen atoms are hidden for clarity.

A model orbital energy diagram proposed by Gray et al. [8] for the [Rh(CNPh)_4_]_2_^2+^ complex (Figure 5A) has the metal d_z_^2^–d_z_^2^ bonding and antibonding orbitals to be HOMO‐1 and HOMO, respectively, while the p_z_–p_z_ bonding and antibonding orbitals are LUMO and LUMO + 1, respectively. Such an ordering remains unchanged across the Co(I)–Ir(I) series, as exemplified by the Rh(I) Dimen complex shown in Figure 5B [9, 10, 13, 15, 16, 17, 18]. For that reason, it is a widely accepted model for d^8^–d^8^ configuration interaction. Moreover, comparison with the corresponding isolated metal ions, where these frontier orbitals are derived primarily from pure metal *d*‐atomic orbitals, reveals that this typical d^8^–d^8^ orbital arrangement is due to mainly the ligand field.

**FIGURE 6:**
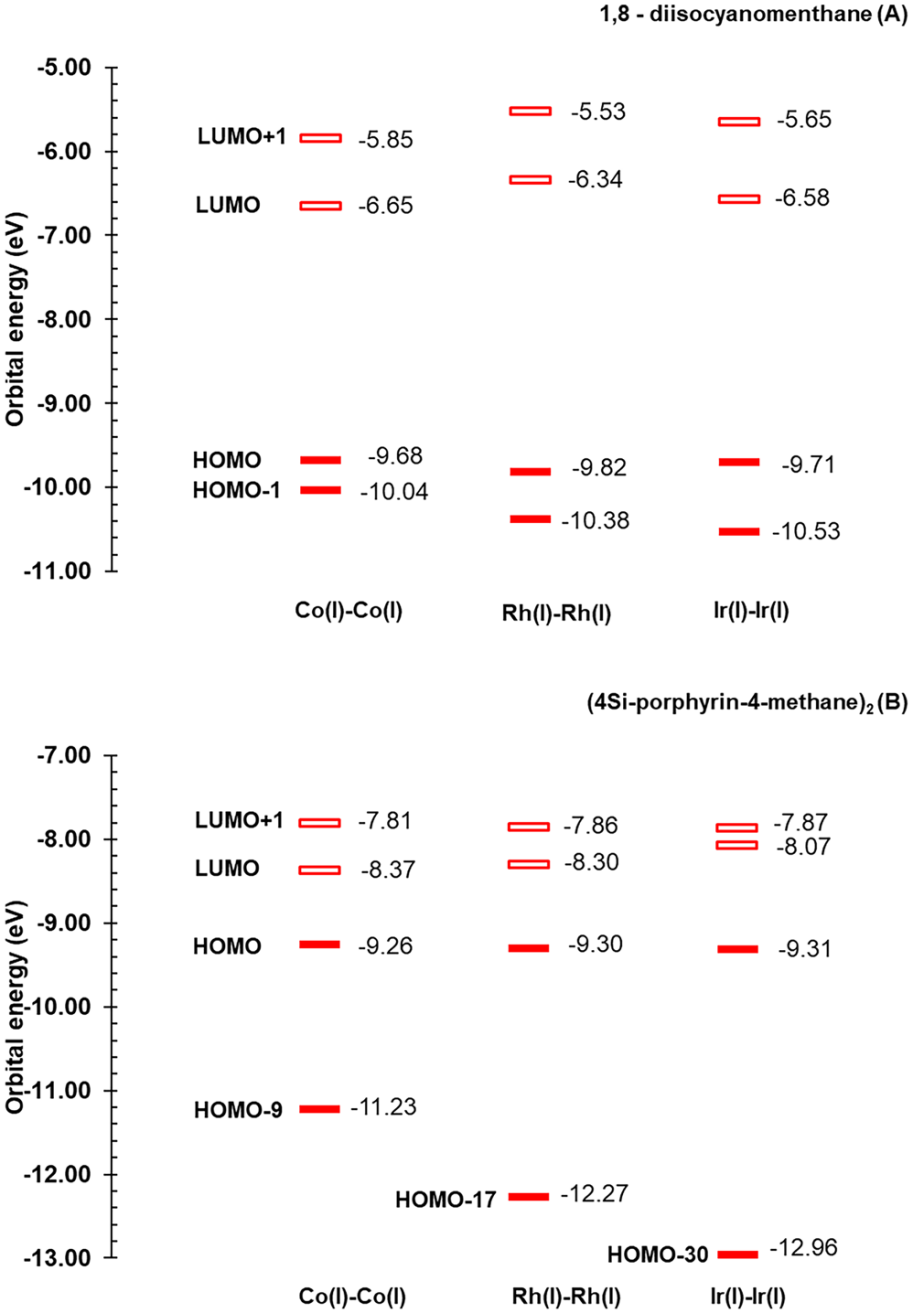
Frontier molecular orbital diagram of Co(I), Rh(I), and Ir(I) complexes with 1,8‐diisocyanomenthane ligand (A) at the singlet state. Frontier molecular orbital diagram of Co(I), Rh(I), and Ir(I) complexes with (4Si‐porphyrin‐4‐methane)_2_ ligand (B) at the singlet state.

In this study, we found that such observed ordering is not universal. In particular, when the ligand field is changed from C donors to N donors as with LOF, not only is MO energy ordering changed dramatically, but also the nature of these frontier orbitals is transformed. As shown in Figure 5C, HOMO is now ligand‐centered (π‐orbitals), while LUMO is the d_z_^2^–d_z_^2^ antibonding orbital instead. More importantly, the d_z_^2^–d_z_^2^ bonding orbital is dramatically stabilized as it is lowered to HOMO‐9 in Co(I), HOMO‐17 in Rh(I) and to HOMO‐30 in Ir(I) (see Figure 6A,B). Note that similar shifts have been reported for P‐donor ligands by Sasakura et al. [14], but their distinct periodic and 3d‐valence properties limit a direct comparison with C‐donor systems as done here.

As discussed above, a sharp contrast is observed between triplet‐dominated metal–metal bonding in Dimen complexes and singlet‐dominated bonding in LOF complexes. This distinction can be rationalized from frontier orbital analysis. In Dimen complexes, the singlet state has a very small M—M bond order because electrons simultaneously occupy both bonding (HOMO‐1) and antibonding (HOMO) orbitals, thus canceling the bonding effect. Partial bonding becomes possible in the triplet state, where an electron is promoted from the antibonding HOMO to a bonding p_z_‐character LUMO.

Conversely, in singlet LOF complexes, HOMO is the bonding from π‐orbitals of the ligand. Excitation to the triplet state promotes an electron from the HOMO to the antibonding d_z_^2^–d_z_^2^ LUMO, effectively reducing the bond order. Consequently, these differences in MO characteristics lead to opposite behaviors: the triplet state in Dimen complexes results in bond shortening, whereas the triplet state in LOF complexes leads to bond lengthening.

### 3.3 A Potential HOMO–LUMO Gap Design

Within the metal–metal interaction framework derived from metal d_z_^2^–d_z_^2^ and p_z_–p_z_ orbital overlap, proposed by Rundle and Miller [44], the magnitude of the band gap in such three‐dimensional periodic systems can be rationalized primarily in terms of the metal–metal distance: shorter metal–metal distances lead to smaller energy gaps, whereas longer distances result in wider gaps [45]. According to Koopmans' theorem (or Janak's theorem [46] in density functional theory), this gap can be reasonably approximated by the HOMO–LUMO energy gap [47]. This approximation is widely adopted in computational studies of molecular and framework materials [48]. Thus, the total density of states (TDOS) plot for a single molecular unit can be used to estimate the electronic trends expected in the corresponding periodic systems.

**FIGURE 7:**
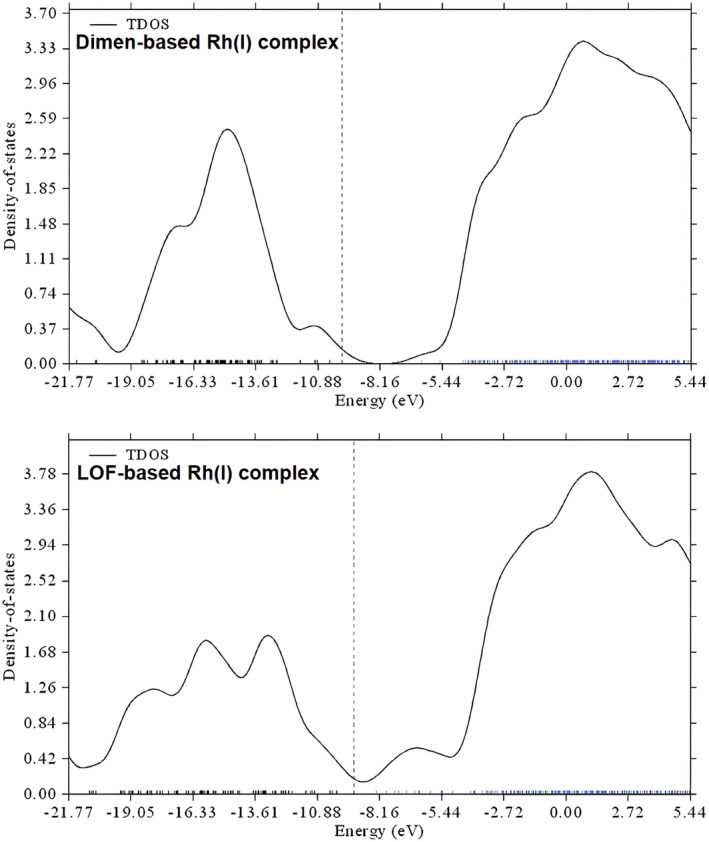
Total density of states (TDOS) for the Dimen‐based (top) and LOF‐based (bottom) Rh(I) complexes. The dashed vertical line denotes the reference energy (HOMO level).

The TDOS plots in Figure 7 show that the Dimen‐based Rh(I) complex (top panel) exhibits a relatively wide energy gap, which can be attributed to a large metal—metal bond distance. On the other hand, the gap in the LOF complexes no longer arises purely from metal d_z_^2^–d_z_^2^ and p_z_–p_z_ interactions. Instead, they exhibit a substantial mixing between metal‐centered d_z_^2^ orbitals and ligand‐based π‐orbitals, leading to a pronounced reduction in the HOMO–LUMO gap of 2.00 eV, even in a single unit cell. Notably, a reduction of this magnitude is typically achieved through extended π‐stacking in conventional systems, suggesting that the LOF architecture may offer a promising strategy for band‐gap engineering.

### 3.4 Comparison of Partial Sigma Bonds in Transition Metals and Carbon‐Cage Biradicals

Table 3 summarizes the similarities and differences between partial covalent σ‐bonds in transition–metal interactions and carbon biradicals. Both systems exhibit long bond distances and partial bond orders, placing them in the concept of *partial covalent bonding*. Carbon biradicals display bond orders in the range of 0.4–0.5 with elongated distances from 2.6 to 5.9 Å, whereas d^8^–d^8^ metal interactions cover a broader bond‐order window (0.3–0.7) from 2.7 to 3.1 Å.

**TABLE 3:** Comparison of partial sigma bonds in transition metals and carbon cage biradicals.

Partial sigma bond type		d^8^–d^8^ transition metals	Carbon biradicals
Bond order		0.3–0.7	0.4–0.5
Bond distance		2.7–3.1 Å^a^	2.6–5.9 Å
Formation state	Singlet	Yes	Yes
	Triplet	Yes	No
Orbital character		d_z_^2^–d_z_^2^ and p_z_–p_z_	p_z_–p_z_
Secondary effect		Ligand field	Hyperconjugation

^a^ The range of bond distance is restricted by organic framework.

A distinct difference arises in the spin‐state dependence. In carbon cages, partial bonding is strictly confined to the singlet state. By contrast, in d^8^–d^8^ systems, both singlet and triplet states can support partial bonding, with the determining factor being the ligand field.

In C‐donor isocyanide (Dimen) complexes, the frontier orbitals follow the classical model: the HOMO is the antibonding metal d_z_^2^ orbital, while the LUMO has the bonding p_z_ character. This arrangement leads to the triplet state being more effective in metal–metal bonding. Conversely, Metal–LOFs have a significant orbital mixing; the HOMO becomes ligand‐centered (π‐orbitals), while the antibonding d_z_^2^ shifts to the LUMO. Crucially, the corresponding bonding d_z_^2^ orbital is deeply stabilized below the frontier region. This configuration explains why the singlet state is dominant for metal–metal bonding in the Metal–LOF systems.

Moreover, the secondary stabilizing effects differ in carbon systems; hyperconjugation from adjacent C_α_—H bonds enhances partial bonding. In contrast, in metal systems, the ligand field effect reorganizes the frontier orbital ordering, resulting in changing characters of frontier orbitals and modulates HOMO–LUMO gaps, thereby determining which spin state dominates the metal–metal interaction.

Taken together, these comparisons reveal that partial σ‐bonding is a general phenomenon extending across both nonmetals and transition metals. This underscores that partial covalency is not element‐specific, but a universal bonding picture shaped by orbital symmetry and environment.

## 4 Conclusion

This work proves that partial σ‐covalent bonding, a general electronic phenomenon extending beyond main‐group biradicals to d^8^–d^8^ transition‐metal systems, is possible. By using dispersion‐corrected DFT (B3LYP‐D3/def2‐TZVP for transition metals and 6‐31+G(d) for other atoms) with Wiberg Bond Index and Frontier Molecular Orbital analyses on Co(I), Rh(I), and Ir(I) complexes, we demonstrate that partial σ‐bonds are sensitive to ligand identity and orbital symmetry.

We find that while shorter M—M distances correlate strongly with larger Wiberg bond orders in general, but this bond index is affected by both metal—metal bond distance and electronic‐state factors. Also, the stability of the singlet versus triplet state arises from a competition between HOMO–LUMO ligand‐field splitting and spin‐orbit coupling. In the strong‐field isocyanide (C‐donor) environment, the HOMO has d_z_^2^–d_z_^2^ antibonding character while the LUMO derives from bonding p_z_ orbitals, resulting in triplet‐dominant metal–metal bonding, (WBO_Triplet_ = 0.4; d_M‐M_ = 2.9 Å). However, the large orbital splitting caused by the strong ligand field energetically disfavors the triplet state. In contrast, the N‐donor Lantern Organic Framework causes a frontier‐orbital reordering, in which the HOMO becomes ligand‐centered and bonding, while the LUMO corresponds to a d_z_^2^–d_z_^2^ antibonding orbital. This reorganization permits substantial partial σ‐bondings in both spin states (WBO_Triplet_ = 0.3 with d_M‐M_ = 3.1 Å and WBO_Singlet_ = 0.7 with *d*_M‐M_ = 2.7 Å). Consistent with the weaker ligand‐field splitting, LOF environment yields smaller HOMO–LUMO gaps, generally favoring the triplet state, but re‐stabilize the singlet in the Ir–LOF complex. Beyond partial bonding, strong mixing between metal d_z_^2^ and ligand π‐orbitals in M‐LOFs produces a pronounced HOMO–LUMO gap reduction of approximately 2.0 eV compared to conventional systems, highlighting a viable pathway for single‐unit‐cell band‐gap engineering without the need for extended lattice stacking.

Overall, partial σ‐bonding in transition metal complexes is identified not as a fixed property between two elements, but as a feature controlled by both the ligand field and electronic structure in transition metals. These results highlight how electronic tuning via ligand design can modulate metal–metal interactions, providing guiding principles for developing mixed‐valence materials and metalloligand frameworks.

## Author Contributions

**Lam H. Nguyen:** writing – original draft, conceptualization, Investigation. **Thanh N. Truong:** writing – original draft, conceptualization, supervision.

## Funding

The authors have nothing to report.

## Conflicts of Interest

The authors declare no conflicts of interest.

## Data Availability

The data that support the findings of this study are available from the corresponding author upon reasonable request.
